# Orodispersible Films: A Delivery Platform for Solid Lipid Nanoparticles?

**DOI:** 10.3390/pharmaceutics13122162

**Published:** 2021-12-15

**Authors:** Denise Steiner, Jakob F. Emmendörffer, Heike Bunjes

**Affiliations:** 1 Institut für Pharmazeutische Technologie und Biopharmazie, Technische Universität Braunschweig, Mendelssohnstraße 1, 38106 Braunschweig, Germany; j.emmendoerffer@tu-braunschweig.de (J.F.E.); heike.bunjes@tu-braunschweig.de (H.B.); 2 Zentrum für Pharmaverfahrenstechnik (PVZ), Technische Universität Braunschweig, Franz-Liszt-Straße 35a, 38106 Braunschweig, Germany

**Keywords:** solid dosage form, poorly water-soluble drug, triglycerides, dual centrifugation, nanotechnology

## Abstract

To overcome the poor bioavailability observed for many newly developed active pharmaceutical ingredients (APIs), an appropriate formulation strategy is necessary. One approach is the formulation of these substances in solid lipid nanoparticles and their further processing into solid dosage forms. A promising and innovative oral delivery platform could be orodispersible films (ODFs). ODFs were already investigated more closely, e.g., for the administration of API nanoparticles, and proved their suitability for this formulation approach. The current study was aimed at investigating if the HPMC (hydroxypropyl methyl cellulose) film matrix is also suitable to serve as an appropriate delivery platform for solid lipid nanoparticles. Dependent on the type of triglyceride nanoparticles embedded in the film matrix and the formulation of the lipid particles, lipid contents of up to 54 wt.% could be realized in the film matrix without the loss of the nanoparticulate state. Good mechanical properties were confirmed for these films by determining the tensile strength as well as the elongation before breakage. Interestingly, processing of a lipid suspension into this solid dosage form led to a significantly reduced transformation of the lipid particles from the metastable α- into the stable β-polymorph. This could prove very beneficial when the lipid particles are loaded with APIs.

## 1. Introduction

An often discussed issue with newly discovered active pharmaceutical ingredients (APIs) is their poor water solubility, which often results in a very low bioavailability [1]. Thus, the formulation of these substances is quite challenging and various technologies have been developed to overcome these issues [2,3]. One promising approach, especially for APIs with a very high lipophilicity, is their formulation in lipid dispersions, such as lipid nanosuspensions [4]. One of the greatest advantages of lipid nanodispersions, which clearly contributes to the overall safety of the drug product, is their good biocompatibility. This enables many routes of administration, such as peroral, parenteral or dermal [5,6]. While formulations of solid lipid nanoparticles are mainly being investigated with regard to parenteral or dermal application, studies indicate that most patients prefer a drug product for peroral use [7]. Since a successful therapy strongly depends on the regular intake of the medication and accordingly a high patient’s adherence, a more patient-centric formulation of the lipid suspensions would be advantageous.

Various studies indicated that the oral bioavailability of APIs can be increased when they are formulated in lipid nanoparticles [8,9,10]. It was also reported that lipid formulations can reduce food effects [11]. However, the exact mechanism underlying the improvement in oral bioavailability is still controversial, as it may be due to a combination of different effects [8]: It is assumed that formulation of the API in a lipid matrix may have a positive effect on the chemical and enzymatic degradation and inactivation by digestive enzymes in the gastrointestinal tract. Furthermore, the improved solubility in the presence of a lipid matrix enables a higher drug load of the formulation and thus higher effective doses. However, the predominant mechanism to enhance the bioavailability of APIs formulated in lipid dispersions was assumed to be a positive interaction with the lipolysis pathway in the gastrointestinal tract. While digested, the lipid particles are metabolized to surface active monoglycerides that form mixed micelles with the bile salts, which can enclose drug molecules. From these mixed micelles, the API is assumed to be directly absorbed together with the lipid. The secretion of bile salts may additionally enhance the solubilization of orally applied, poorly water-soluble APIs, and thus enable their direct absorption [9,10,12,13]. 

Against the background of the positive effect of orally applied lipid formulations on the bioavailability of poorly water-soluble APIs, several studies have already addressed the further processing of solid lipid nanoparticle dispersions into solid dosage forms. One possibility is spray-drying of the lipid suspensions. To prevent particle agglomeration, matrix materials, such as lactose, mannitol or carrageenan, were added to the suspensions as matrix-forming materials and the formulations were diluted with water and/or organic solvents. While some of these studies mainly concentrated on the characterization of the resulting lipid-containing powders [14], others also considered the physical state of the embedded lipid nanoparticles by redispersing the powders in water or aqueous solutions [15,16]. Depending on the formulation and the preparation of the solid lipid nanoparticles, some studies succeeded in retaining the size of the lipid nanoparticles in the powders. Further studies focused on freeze-drying of the nanoparticle suspensions. Prior to lyophilization, the lipid particles were embedded in a gel matrix to generate a porous structure of the dried formulations. This enabled controlling the release profiles of the APIs loaded in the solid lipid nanoparticles, depending on the applied gel-forming material [17,18].

Drying the lipid nanosuspensions at more moderate temperatures is possible when embedding them in a film-forming polymer upon preparation of orodispersible or buccal drug delivery systems. Orodispersible or buccal films are thin, polymer-based dosage forms. Buccal films adhere to the oral mucosa when placed in the mouth, thereby supporting transmucosal absorption of the API. Orodispersible films (ODFs) disintegrate within seconds on contact with aqueous media and allow the API formulation to be swallowed with saliva [19]. The major advantage of both the solid dosage forms is that they do not have to be swallowed as a whole, e.g., tablets or capsules. Depending on age and health status, patients may have problems swallowing larger monolithic dosage forms [20]. Furthermore, these films enable a very flexible dosing and have the potential to overcome the limitations of most of the conventional solid dosage forms on the market even though the formulative space of ODFs (<200 mg) is quite limited [21]. A few studies already focused on the embedding of solid lipid nanoparticles in film formulations, mainly mucoadhesive buccal films. Jones et al. successfully loaded solid lipid nanoparticles with didanosine and administered these via the buccal route to avoid the fast degradation of the API in the gastrointestinal tract. There, the film matrix was made of Eudragit^®^ RS 100, triethyl citrate and hydroxypropyl methylcellulose (HPMC) to enable buccal drug delivery. In vitro permeation experiments with a porcine buccal mucosa indicated that a higher cumulative amount of API permeated per unit surface area when the API was embedded in the lipid particles [22]. Another study investigated film formulations with coumarin 6-loaded lipid nanoparticles embedded in a HPMC film-forming matrix to evaluate the critical quality attributes of the film preparation as well as mucoadhesion of the films. Furthermore, the permeability of the model drug from different formulations was investigated with a monolayer of differentiated HT29-MTX cells [23]. So far, only one study focused on the embedding of lipid microparticles in ODFs. There, the API melatonin was dispersed in tristearin or hydrogenated castor oil and the prepared lipid microparticles were embedded in a maltodextrin film matrix [24].

The current study evaluated the possibility to administer solid lipid nanoparticles in an orodispersible film matrix. For this, an appropriate particle formulation is crucial to preserve the small size of the lipid nanoparticles upon drying and redispersion of the film in water. To make the best possible use of the limited volume of the films with the intention to enable the highest possible API load in future studies, different concentrations of triglyceride particles were embedded in the film matrix. Although high particle loads were aimed for, a major focus was to achieve films with a good quality to enable appropriate handling by the patient. Another characteristic that was of interest in this study was the polymorphic form of the lipid nanoparticles. Solid triglyceride nanoparticles undergo a time-dependent polymorphic transformation depending on the lipid particle formulation [25,26,27,28]. Former studies reported indications that the transformation from the metastable α-polymorphic form into the stable, more ordered crystal structure of the β-polymorph could result in lower API-loading capacities [29,30]. Thus, it was of interest whether an embedding into the rigid film structure would affect—and potentially suppress—the polymorphic transition. 

## 2. Materials and Methods

### 2.1. Materials

The three different triglycerides tristearin (Dynasan^®^ 118), tripalmitin (Dynasan^®^ 116) and trimyristin (Dynasan^®^ 112) were used in this study. All were from Hüls AG/Cremer Oleo (Witten, Germany) and kind gifts from the manufacturer. For lipid particle stabilization the following stabilizers were investigated: hydroxypropyl methyl cellulose Pharmacoat 606 (HPMC; Shin-Etsu Chemical Eo., Tokyo, Japan; kind gift from Harke Pharma, Mühlheim, Germany), polyvinylpyrrolidone Kollidon^®^ 30 (PVP; BASF, Ludwigshafen, Germany), vinylpyrrolidone-vinyl acetate copolymers Kollidon^®^VA 64 (KVA 64; BASF, Ludwigshafen, Germany), polyvinyl alcohol Mowiol 3-83 (PVA, Kuraray Europe GmbH, Hattersheim, Germany; kind gift from the manufacturer), poloxamer 188 (P188, BASF, Ludwigshafen, Germany; kind gift from the manufacturer), polysorbate 80 (Tween80, Caesar & Loretz GmbH, Hilden, Germany) and sodium dodecyl sulphate (SDS, Roth, Karlsruhe, Germany). For the preparation of ODFs, the film-forming polymer HPMC and the plasticizer glycerol (Roth, Karlsruhe, Germany) were used. All formulations were prepared with distilled water. 

### 2.2. Stabilizer Screening by Dual Centrifugation

The stabilizer screening was performed with a modified dual centrifuge, ZentriMix 380R (Andreas Hettich GmbH & Co KG, Tuttlingen, Germany). In comparison to the other dual centrifuges of this type, the used model is equipped with a heating coil at the bottom of the process chamber to realize temperatures up to 60 °C. This enables the processing of lipids that are solid at room temperature, as reported before [31]. 

The screening experiments were performed in 2 mL DC-Twist-Top-vials from Andreas Hettich GmbH & Co KG (Tuttlingen, Germany, kind gift from the manufacturer) and all formulations were prepared and processed twice. For all screening experiments, the lipid content in the formulation was 10% (all concentrations given in this study are *w*/*w*) and the total formulation amount in the vials was 1 g. This experimental setup was chosen based on experience gained with the dual centrifuge in a former study [31], where good results were achieved with a lipid content of 10% and it was assumed that this lipid content also enables a scale-up to a high-pressure homogenizer. The stabilizer solution was prepared by dissolving the stabilizer under investigation in water at a concentration of 10% and, if applied, additionally 1% SDS (both concentrations refer to the total sample weight). The stabilizer solution and the unmolten lipid were put into the vial at room temperature. To enable the appropriate mechanical stressing of the formulation during processing, yttrium-stabilized zirconium dioxide grinding beads with a size between 0.5 and 0.7 mm (Sigmund Lindner GmbH, Warmensteinach, Germany) were added. The beads had a spherical shape and a material density of ρ = 6.05 kg dm^−3^. The filling ratio of the grinding media was 30%, referring to the volume of the vials.

Before inserting the vials into the dual centrifuge, the device was pre-heated to 60 °C for 30 min. Afterwards, the samples were placed in the sample holder and stressed for 10 min with a rotor speed of 2350 rpm (revolutions per minute). The particles were then crystallized by cooling the formulation in the vials in a fridge down to 5 °C for 120 min. Samples were stored at room temperature before characterization. 

### 2.3. Preparation of Lipid Dispersions and Orodispersible Films

The lipid dispersions to be embedded in the ODFs were prepared by high-pressure homogenization. The formulations under investigation contained 10% lipid, 5% stabilizer and 0.5% SDS, and the formulation stabilized only with the surfactant contained 10% lipid and 0.5% SDS. The stabilizer concentration was chosen based on experience gained in former studies with triglyceride formulations, such as by Goeke et al. [32]. Before high-pressure homogenization, both phases were pre-heated to approx. 85 °C and a pre-mix emulsion was prepared by mixing the molten lipid phase with the aqueous solution containing the dissolved stabilizers and SDS. The pre-formulation was homogenized with a T25 digital ULTRA TURRAX^®^ (IKA, Staufen, Germany) for 2 min at 13,000 rpm. Afterwards, it was high-pressure homogenized at 800 bar in 10 cycles with a Microfluidizer M110-P (Microfluidics, Westwood, MA, USA). The nanoparticles were crystallized by incubating the dispersions in a fridge at 5 °C for 120 min. Afterwards, the samples were stored at room temperature before they were further analyzed or processed to ODFs.

In order to manufacture ODFs, a film casting suspension was prepared containing the lipid nanoparticle suspension and a film-forming matrix solution. The matrix solution was prepared by adding 10% HPMC in water at approx. 80 °C. The solution was stirred and cooled down to room temperature. Afterwards, 3.3% glycerol (all data referred to the total matrix solution weight) was added to the solution and stirred for at least 8 h until no air bubbles remained in the solution. The lipid content in the ODFs was varied by mixing the matrix solution and the lipid suspension at different ratios. The homogenization of the film-casting suspension was performed with an ULTRA TURRAX^®^ for 1 min at 10,000 rpm. After degassing at ambient conditions for approx. 2 h, 9 g of the film-casting mass was poured in a glass Petri dish with a diameter of 10 cm and dried at room temperature. 

For comparison purposes a placebo film was prepared. Similar to the lipid-containing film-casting mass, HPMC (10%) was dissolved in 80 °C warm water and stirred while cooling down to room temperature. Afterwards, glycerol (3.3%) and SDS (0.5%) were added to the solution and homogenized for 8 h. The film-casting mass was also cast in Petri dishes and dried at room temperature. 

After approx. 24 h, all films were removed from the Petri dishes and stored in plastic bags at room temperature until characterization. The ODFs included in the stability study were stored in the plastic bags in a climatic chamber at 20 °C and 40% relative humidity. The lipid contents in the ODFs were calculated according to the amount of applied lipid suspension, formulation stabilizers and film-forming matrix material.

### 2.4. Particle Size Analysis

The particle sizes of the original suspensions as well as of the lipid particles redispersed from the ODFs were measured by laser diffraction (LA-960, Horiba Scientific, Kyoto, Japan). Lipid nanosuspensions were measured one day after their preparation. The sizes of the lipid particles embedded in the film matrix were measured after redispersion of 4 cm^2^ ODF in 0.9 mL water for 1 h. All samples were diluted in distilled water to achieve an appropriate particle concentration for the analysis before measurement. Three measurements were performed of each sample and the volume distribution was calculated according to the Mie theory-based evaluation model. The following refractive indices were assumed: lipid particles 1.46 (absorption index of 0.01) and water 1.33.

In order to describe the width of the particle size distribution, the span was calculated for selected samples according to Equation (1):

(1) $$span=\frac{x_{90}-x_{10}}{x_{50}}$$

### 2.5. Physical Characterization of ODFs 

The mechanical properties of the films were measured with a material testing machine from Zwick GmbH (8136/20N, Ulm, Germany). Six samples were measured for each film formulation and prepared by cutting the ODFs into pieces of 5 mm × 35 mm. Before the measurement, the film thickness of each sample was determined at two positions with a micrometer screw (accuracy 0.001 mm). During testing, the films were held between two grips and stressed at a clapping length of 25 mm with a testing speed of 5 mm min^−1^ until rupture. The tensile strength was calculated from the maximum force before rupture and the mean film thickness of each sample. Additionally, the elongation before breakage was considered as physical measurand to assess the brittleness of the ODFs. The mean values as well as the standard deviations were calculated for all samples.

The disintegration time of the films was analyzed with the optimized SFaB (slide frame and ball) device according to Speer et al. [33]. Before disintegration measurements, the film thickness of the samples (30 × 40 mm) was determined at four different positions and the medium film thickness was calculated. Then the samples were fixed in a frame and 0.9 mL water as well as a stainless-steel ball (size 10 mm and weight 4 g) were placed on top of the ODF. To fix the position of the ball during measurement, an insert was placed on top of the experimental setup. The time measurement was started when the ball was placed on the film surface on top of the water layer and stopped when the ball fell through the ODF to the bottom of the measurement device due to film disintegration. To enable a better comparison of the disintegration times of the various formulations the specific disintegration time was calculated in this study by setting the disintegration time in correlation with the film thickness. 

### 2.6. Differential Scanning Calorimetry 

Differential scanning calorimetry (DSC) was performed to characterize the thermal behavior of lipid nanosuspensions or lipid particles embedded in the ODF matrix. A Mettler Toledo DSC 1 STARe system with FRS5 sensor (Mettler Toledo, Gießen, Germany) was used. Samples were weighed into 40 µL aluminum crucibles and cold welded. When lipid suspensions were analyzed, 18 µL were weighed into the crucibles, while for ODFs, samples with a diameter of 4 mm were stamped out of the films and three films were placed on top of each other (yielding a total sample weight of appr. 5 mg). The experiments were performed at a heating rate of 10 °C min^−1^ starting at a temperature of 25 °C up to 85 °C.

### 2.7. Viscosity Measurement

The viscosity of the lipid nanosuspensions was measured with a rotational viscometer HAAKE^TM^ RheoStress 6000 (Thermo Fischer Scientific, Waltham, MA, USA) with the double gap Searle system DG41. The measurements were performed with a 10 mL suspension at 25 °C and shear rates between 0.1 s^−1^ and 1000 s^−1^ (gap height 5.1 mm). The dynamic viscosity is given independent of the shear rates, because the formulations displayed Newtonian flow properties for the applied shear rates. 

### 2.8. Scanning Electron Microscopy

Images were taken of a selected ODF sample with the scanning electron microscope (SEM) Helios G4 CX (FEI Deutschland GmbH, Frankfurt, Germany). A cross section of the film was prepared and sputtered with gold at 5 mA for 4 min in a Balzers Union SCD 030 sputter device before measurement. The image was taken in high vacuum with a voltage of 1.00 kV.

## 3. Results and Discussion

### 3.1. Screening for a Lipid Nanoparticle Formulation 

**Figure 1:**
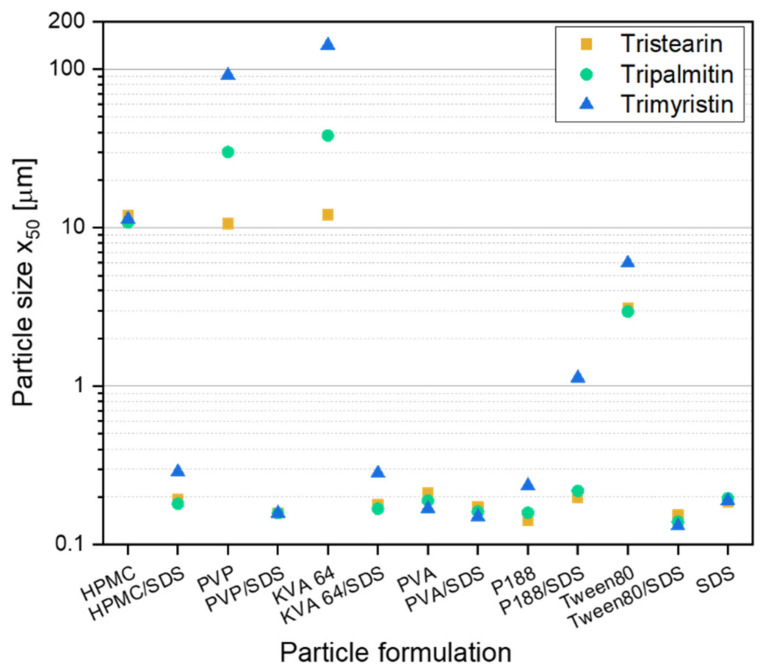
Results of the screening experiments in a dual centrifuge (10 min at 60 °C and 2350 rpm) with different stabilizer formulations for the stabilization of particles from the solid lipids tristearin, tripalmitin and trimyristin (10% lipid in formulation).

Before embedding lipid nanoparticles in ODFs, an appropriate particle formulation was needed to prevent agglomeration during the further processing of the lipid nanosuspensions into solid dosage forms. In former studies lipid dispersions were mainly investigated for parenteral administration. This route clearly reduces the choice of applicable additives that may be used as stabilizers against agglomeration in the suspension. However, when the suspension is to be further processed into an oral dosage form a larger variety of stabilizers, such as polymers or surfactants, which have not been used for lipid particle stabilization so far, can be taken into consideration. In this study, different stabilizers were chosen with and without the additional presence of the surfactant SDS to formulate lipid nanoparticles from the three different triglycerides tristearin, tripalmitin and trimyristin. A stabilizer screening was performed to evaluate which formulation can stabilize the lipids against coalescence during manufacturing and against agglomeration after the recrystallization of the liquid lipid nanoparticles. The screening was performed with a heatable dual centrifuge. This enabled processing in small batches (1 mL) and the simultaneous investigation of up to ten formulations. The resulting particle sizes, x_50_, are shown in Figure 1. To ensure the presence of enough stabilizer in the formulations for the occupation of the newly created lipid surfaces (10% lipid content in the formulation, all concentrations in this study are *w*/*w*), a polymer concentration of 10% and, if present, a SDS concentration of 1% (referred to the total formulation) were chosen. 

Particles larger than 1 µm were mainly detected in formulations only stabilized with the polymer. Only in formulations with PVA and P188 particles with sizes below 300 nm were obtained without the presence of SDS. When SDS was additionally added to the polymer (or Tween 80) solutions, smaller lipid particles were achieved for the suspensions with only one exception (formulation P188/SDS). This result is not unexpected because the suspensions formulated only with SDS (1% SDS in total formulation) achieved particle sizes below 200 nm for all three lipids under investigation. In general, it was assumed that a stable particle system had been obtained when the particle sizes were 300 nm and below. 

There was no clear trend concerning differences in particle sizes between the three lipid types when only the successfully stabilized formulations (particle sizes ≤ 300 nm) were considered. It looks like that for some formulations, the trimyristin suspension could not be stabilized as sufficiently as the formulations with tripalmitin or tristearin. Thus, the following studies regarding the further processing of the lipid suspensions into solid dosage forms were performed with tristearin. 

To evaluate the possibility to embed solid lipid particles in a HPMC film matrix without the loss of their nanoparticulate properties, the emulsification process for the successful, SDS-containing formulations was first scaled-up from dual centrifugation to high-pressure homogenization and the formulation was slightly adjusted. While the lipid concentration of 10% in the formulation was kept constant, the polymer and SDS concentration was reduced to 5% and 0.5% (referred to the total formulation), respectively. The lipid suspension stabilized only with SDS had a total surfactant concentration of 0.5%.

**Figure 2:**
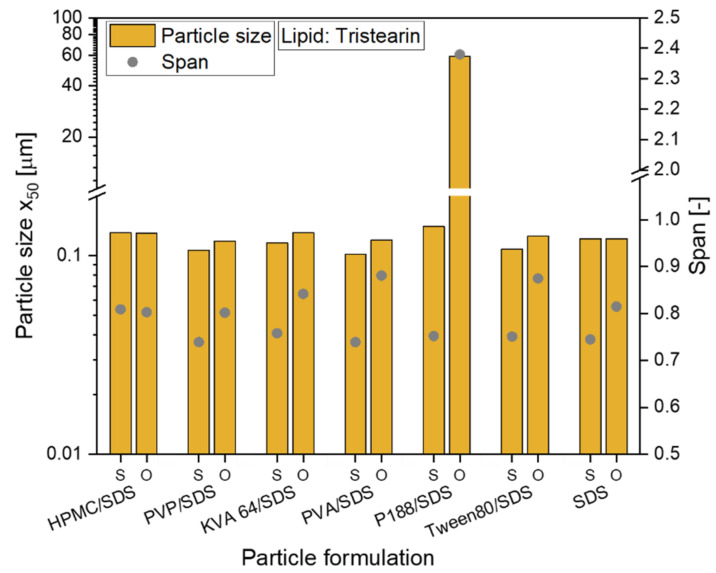
Investigation of the redispersiblity of differently formulated tristearin nanosuspensions from the film matrix; particle size x_50_ and span of the size distribution are given for original suspensions (S) and for redispersed lipid particles from ODFs (O).

The preparation of all formulations could be scaled-up from dual centrifugation with a sample volume of 1 mL to high-pressure homogenization (sample volume 50 mL). For all tristearin suspensions, particle sizes (x_0_) between 102 and 140 nm were obtained (see Figure 2, abbreviation S). The tristearin nanoparticles were then embedded in the film-forming matrix with a lipid particle content of usually 0.29. When the particles were stabilized only with SDS, the lipid content in the ODF was slightly higher (lipid content 0.33). To evaluate if particle agglomeration occurred during film preparation, the films were redispersed in water and the sizes of the lipid particles were measured. With only one exception, the particles could be redispersed with no loss of their nanoparticulate properties and a size increase of by maximum 24 nm (see Figure 2, abbreviation O). Only the tristearin particles formulated with P188/SDS showed a clear particle agglomeration after film redispersion. So far, it could not be clarified what kind of interactions caused this strong particle agglomeration. 

To also enable a comparison of the particle size distributions, the span was calculated for all formulations and is also given in Figure 2. While for the formulation with HPMC/SDS the size distribution did not change at all, an increase of the span was detected for the other formulations. Having a closer look at the actual increase of the span, it was approx. 0.06 to 0.14 higher for the particles after redispersion from the ODFs than for the original lipid suspensions (except for the P188/SDS formulation). In general, this low increase was considered acceptable. It demonstrated that almost every tested formulation is suitable to stabilize the tristearin nanoparticles in the HPMC matrix without particle agglomeration. 

Hereinafter, four different formulations were chosen for further investigations: HPMC/SDS, PVA/SDS, PVP/SDS and SDS. 

### 3.2. Influence of Lipid Content on ODF Properties 

#### 3.2.1. Redispersibility of Lipid Particles from Films 

**Figure 3:**
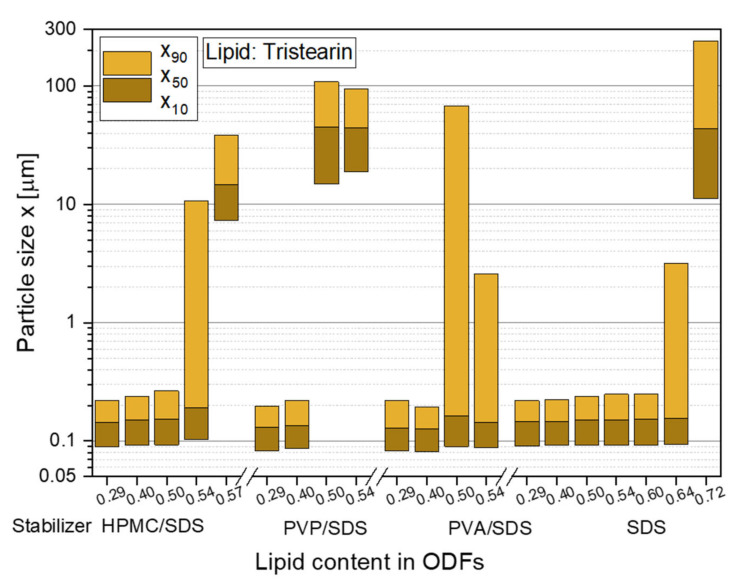
Particle size distributions of solid lipid tristearin nanoparticles redispersed from ODFs with various lipid contents and formulated with different stabilizers. The particle size distributions of the original lipid nonosuspensions are shown in Figure 2.

To realize high API loads in the ODFs in future studies, the lipid contents in the films were stepwise increased to identify the maximum lipid particle load, which did not lead to particle agglomeration in the film matrix. The first feasibility study with tristearin already revealed that a lipid content of 0.29 in the ODFs did not significantly increase the particle sizes after redispersion in water compared to the initial lipid suspension (see Figure 2). The same behavior was observed for a lipid load of 0.40 in the films, independent of the chosen particle formulation. However, with a further increase of the lipid content to 0.50 an influence of the particle stabilization on the particle sizes after film redispersion was determined (see Figure 3).

The stabilizer combination PVP/SDS seemed to be least suitable for the stabilization of the particles in the HPMC film matrix. Large agglomerates with sizes of more than 1 µm were redispersed from the ODFs when the lipid content was 0.50 and higher. While it was assumed that the particle formulation mainly had an influence on the agglomeration tendency of the particles, a poor film quality was also detected for these films. They could hardly be removed from the glass Petri dishes and all ODFs had a high brittleness independent of the lipid content. This leads to the assumption that the polymer and surfactant for the particle stabilization was not only located at the lipid particle surfaces but was also dispersed in the film-forming formulation where it influenced the film quality. 

Better films qualities were achieved with the particle formulation PVA/SDS but x_90_-values of over 2 µm were measured after film redispersion as soon as a lipid content of 0.50 and higher was embedded in the ODFs. A lipid load of up to 0.50 with only a slight increase in the particle sizes was obtained when HPMC was used as stabilizer (in combination with SDS) and as film-forming polymer. With lipid contents of up to 0.60 and no loss of the nanoparticulate properties, the highest amount of lipid nanoparticles could be embedded in the ODFs when the lipid suspension was formulated only with the surfactant SDS. In general, the overall composition of the ODFs formulated with only SDS did not differ from the films stabilized with HPMC/SDS but a significantly higher amount of lipid could be embedded in the ODFs without any particle agglomeration. It is assumed that this difference is caused by the different viscosities of the lipid suspensions and consequently the film-casting masses. HPMC was used as film-forming material in this study and, thus, had a clear influence on the viscosity of the formulation when dispersed in water. Due to the presence of HPMC in the initial lipid suspension, its viscosity was higher (suspensions: η_HPMC/SDS_ = 103 mPas and η_SDS_ = 14 mPas) and the homogenization of the suspension with the HPMC solution is assumed to have been less efficient especially with a lipid content of more than 0.50 in the formulation. The lower viscosities of the SDS-stabilized formulation seem to be beneficial for a more homogenous distribution of the lipid nanoparticles in the film-casting suspension. 

Lipid contents in the films of over 0.50 indicate a promising approach to administer an appropriate dose of API to a patient with only one standard-sized ODF. Further investigations regarding the film properties of films containing the two most promising particle formulations (with HPMC/SDS and SDS) were performed and are shown below (Section 3.2.3. and Section 3.2.4.).

#### 3.2.2. Polymorphic Form of Lipid Particles in ODFs 

**Figure 4:**
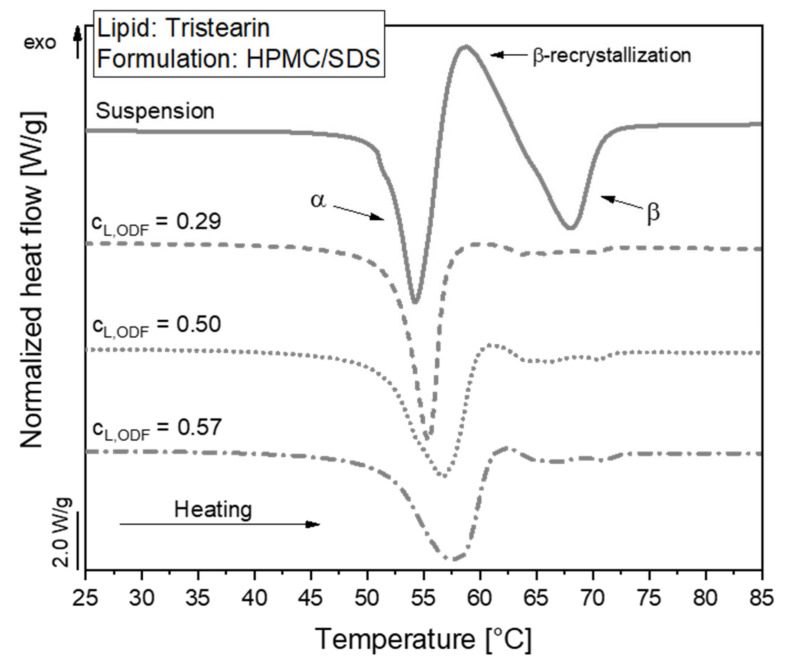
DSC melting curves of lipid nanoparticles stabilized with HPMC/SDS as suspension (lipid content 0.1) and embedded in ODFs with different lipid contents (c_L,ODF_) measured one day after film preparation. To facilitate visual comparison of the curves, the heat flows were normalized to the sample mass and the lipid concentration of the respective formulation.

The polymorphic form of the tristearin formulation stabilized with HPMC/SDS was analyzed by DSC. The heat flow obtained during the melting process of the suspension and corresponding ODFs is shown in Figure 4. 

The melting curve of the particles in the suspension reflects a typical behavior of tristearin nanoparticles, as described earlier [28,34]. When the sample is heated, the particles melt first from the metastable α-polymorphic form, as illustrated by the endothermic peak at approx. 54 °C. Shortly afterwards, an exothermic event is detected at approx. 58 °C. This indicates a recrystallization of the previously melted α-from particles into the stable β-polymorphic form. At approx. 67 °C, the endothermic event indicates the melting of the β-form particles. 

A large melting signal of the α-form was also observed in the ODF samples. This indicates that the polymorphic form of the lipid particles could be preserved when the nanosuspensions were further processed into the solid films. The subsequent recrystallization and β-melting events were much smaller for the ODF samples as compared to the liquid suspension. The thermal maxima were slightly shifted to higher temperatures and the α-melting endotherms were broader when more lipid particles were embedded in the films. The onset temperature of the peaks as well as the melting enthalpies (normalized to the sample mass and lipid concentration) were, however, not significantly different between the samples under investigation. Thus, it was assumed that the shape of the peaks was influenced by the material surrounding the lipid particles and the experimental setup in the DSC. During the measurement, the aluminum crucibles are heated from the bottom. Due to poorer heat transfer through the solid HPMC matrix and the three films stacked on top of each other in the crucibles compared to the aqueous phase in the suspension, minor differences may be expected in the shape of the curves. Much more detailed investigations would be required to elucidate if the differences in peak shape may also point to subtle alterations in the state of the lipid particles. 

#### 3.2.3. Mechanical Film Properties 

**Figure 5:**
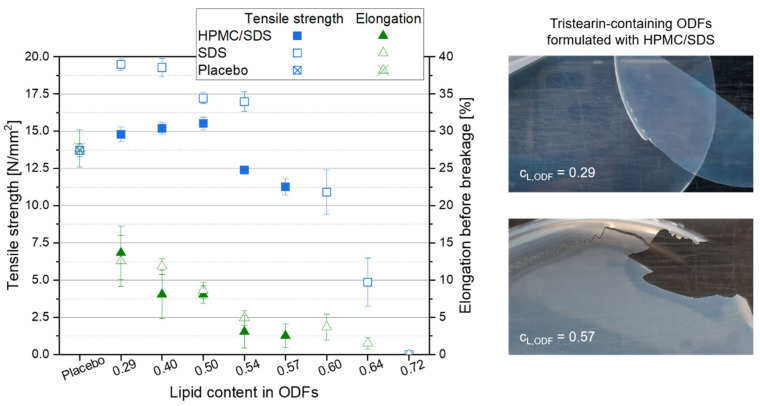
Mechanical properties of tristearin-containing ODFs loaded with various lipid contents as compared to a lipid-free placebo film (left, *n* = 6), and images of ODFs loaded with an HPMC/SDS-formulated tristearin suspension at lipid contents c_L,ODF_ = 0.29 (right, top) and c_L,ODF_ = 0.57 (right, bottom).

High lipid contents in ODFs are very advantageous with respect to drug carrier capacity but also decrease the content of film-forming polymer in the films. The mechanical properties of the films were determined to evaluate which ODFs could still be handled by patients as a whole without breaking or formation of fragments to ensure a correct API dosing. The mechanical film properties “tensile strength” and “elongation before breakage” were considered for this evaluation. While the tensile strength describes the traction that can be applied before the film breaks, the elongation before breakage was assessed to evaluate the brittleness of the films. Lipid-free placebo films were used as reference and the results are displayed in the graph in Figure 5. Compared to the placebo film, embedding of the lipid particles into the film matrix had no major negative effect on the tensile strength of the films up to a lipid content of approx. 0.54. Dependent on the formulation of the lipid particles in the ODFs, the tensile strength even increased when lipid particles were embedded in the HPMC matrix. Compared to the tensile strength of the placebo film (13.7 N mm^−2^), maximum values of 19.5 N mm^−2^ for the SDS-ODFs (lipid content 0.29) and of 15.5 N mm^−2^ for HPMC/SDS-ODFs (lipid content 0.50) were obtained, respectively. With only two exceptions for the SDS-formulated ODFs (lipid contents 0.64 and 0.72), all films had a tensile strength above 10 N mm^−2^, which indicates a high resistance against the applied traction for these lipid-loaded ODFs. Similar tensile strengths were obtained before; e.g., when the nanoparticles were loaded into an HPMC matrix [35]. 

Comparing the tensile strengths of the different film types with each other it is remarkable that the films that contained particles stabilized only with SDS had higher tensile strengths than the films containing HPMC/SDS-stabilized lipid particles, although the overall composition of the films was the same. In general, these films only differed in the time point the HPMC was added to the formulations. When the particles were formulated with HPMC/SDS and a certain portion of the film-forming matrix was already added as particle stabilizer to the formulation, it is assumed that an unknown amount of the HPMC was bound to the surface of the lipid particles together with the SDS to prevent their agglomeration or coalescence. Consequently, it can be assumed that the bound HPMC molecules could not contribute to the formation of the film matrix to the same extent as the HPMC molecules added later in the manufacturing process resulting in lower tensile strengths of the films containing HPMC/SDS-formulated nanoparticles. 

Another important characteristic to describe the handleability of the ODFs is the film elongation before breakage because it gives an impression of the brittleness of the films (see Figure 5). The results show a clear increase in the brittleness as soon as particles were embedded in the film-forming matrix. For both film formulations the elongation before breakage decreased with a higher lipid content in the ODFs. The images of the films (see Figure 5, right) give an impression of how the film’s brittleness influences the handling of the ODF. Even though the elongation before breakage of the films with a lipid content of 0.29 was clearly lower than that of the placebo film (approx. 13.0% vs. 27.7%), these lipid-containing films were flexible and could be removed from the Petri dish after drying without any difficulties. A particle content of 0.57 resulted in an elongation before breakage of only 2.5%. This film could still be peeled off the Petri dish almost in one piece but several cracks indicated a much higher brittleness although the tensile strength of this film was still above 10 N mm^−2^. The handling of the films in this study clearly demonstrated that an elongation before breakage of at least 5.0% is required to allow adequate sample preparation and film characterization. This restricts the maximum lipid content in the films to 0.50 for the HPMC/SDS and 0.54 for the SDS formulation. This is in accordance with the maximum possible particle load in the films at which the nanoparticulate properties of the embedded lipid particles are preserved (see Figure 3). 

#### 3.2.4. Disintegration Time of ODFs 

The specific disintegration time of the films was investigated to evaluate the influence of the particle formulation as well as lipid content in the ODFs on the disintegration behavior. Although there is no regulation in the European Pharmacopeia [36] regarding the allowed maximum disintegration time of ODFs, the maximum disintegration time for orodispersible tablets, 180 s, is often taken as reference [37]. 

The dry film thicknesses of the ODFs under investigation varied between 90 µm and 130 µm, depending on their lipid contents. To enable a comparison of the measured disintegration times, the specific disintegration time (which refers to the film thickness) was calculated and the results are given in Table 1. With only one exception, the specific disintegration times increased with higher lipid contents independent of the particle formulation. This is caused on the one hand by the increasing content of hydrophobic lipid particles in the films and on the other hand by the decreasing fraction of water-soluble matrix material. As a result, the disintegration time increased because of the reduced amount of soluble substances at the film surface. Thus, the water applied to the film surface advanced more slowly into deeper layers of the ODF. Although the total amount of SDS in the film formulations increased with higher particle loads (c_total,SDS_ = 0.0145 for lipid content 0.29 and c_total,SDS_ = 0.0320 for lipid content 0.64 in the ODFs), the influence of the higher surfactant concentration in the films and thus, an improved wettability, seems to be of minor importance in this case. A similar effect on the disintegration behavior was previously observed when poorly water-soluble API nanoparticles were embedded in the polymer matrix [37]. The only formulation that does not fit into this general trend was the ODF prepared with a lipid content of 0.64 (formulation with SDS). Caused by the poor mechanical properties of the films prepared from this formulation, small cracks appeared on the sides of the films during sample preparation, resulting in significantly shorter disintegration times. For the films with a lipid content of 0.72, it was not possible to prepare samples with the required size.

Assuming an average film thickness between 100 µm and 120 µm, the maximum disintegration time of the films with good mechanical quality (see Section 3.2.3, for HPMC/SDS lipid content 0.50 and for SDS lipid content 0.54) did not exceed the disintegration time of 180 s. This means that although high lipid contents were embedded in the film-forming matrix, the films still met the specifications that are usually applied for ODFs. 

#### 3.2.5. SEM Analysis of Embedded Particles 

**Figure 6:**
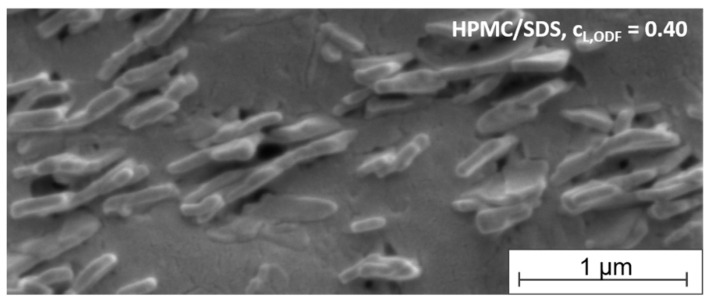
SEM image of the cross-section of an ODF loaded with tristearin particles (lipid content 0.40) formulated with HPMC/SDS.

To get an impression of the arrangement of the tristearin particles in the film-forming matrix, SEM images were taken of the cross-section of an ODF formulated with HPMC/SDS-stabilized tristearin particles at a lipid content of 0.40. As mentioned before, tristearin particles are known to crystallize in mainly two crystal modifications, the α- and the β-modification (Figure 4). Dependent on the modification, the solid lipid nanoparticles can be spherical (α-modification) or platelet-like (β-modification) [38,39]. The image in Figure 6 indicates that single lipid particles could be embedded in the matrix material. This enables a good redispersibility of the lipid nanoparticles in water and preservation of the nanoparticulate properties. 

A closer look at the shape and arrangement of the embedded particles revealed mostly platelet-like particle structures organized in stacks. A stack-like arrangement of triglyceride particles in suspensions has been reported before. Schmiele et al. investigated tripalmitin nanosuspensions in more detail and showed that above a critical particle concentration the platelet-shaped particles initially arranged themselves into small platelet stacks. With further increasing concentrations these stacks merged into nematically ordered domains [40,41]. A similar behavior was observed for trimyristin nanoparticles by this group [42]. According to the SEM image, a similar particle arrangement was found for the tristearin particles in the HPMC matrix in the current study. It seems that the stack-like structures could be preserved in the dried film matrix. 

While the determination of the polymorphic form of the lipid particles by DSC clearly indicated the presence of a high amount of the metastable α-modification (see Figure 4), the platelet-like particle shape in the SEM image rather indicated a β-polymorphic form of the particles. Future studies will have to clarify which process causes the change in the particle shape in this case. 

### 3.3. Influence of Storage Time on Polymorphic Form of Lipid Particles 

**Figure 7:**
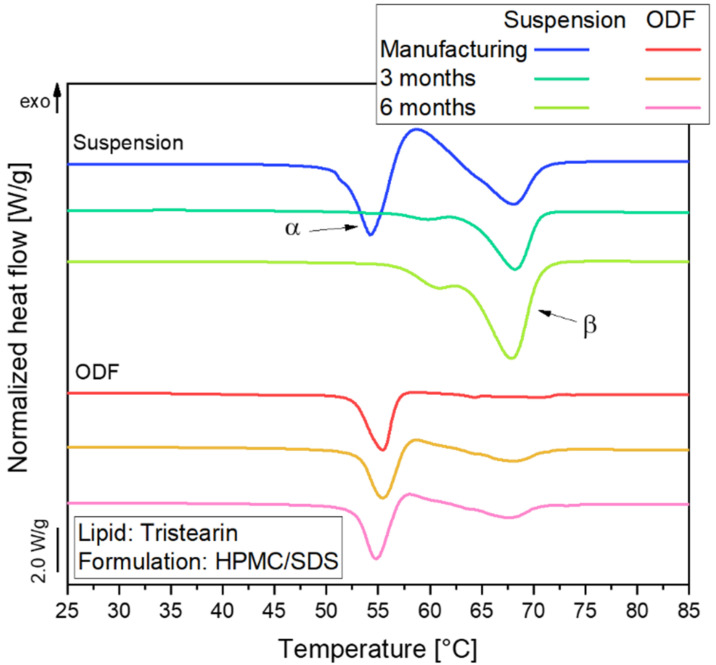
DSC curves of tristearin particles in nanosuspension (lipid content 0.1) and in ODFs (lipid content 0.29) stored over six months at 20 °C and 40% relative humidity; tristearin particles were formulated with HPMC/SDS.

A change in the polymorphic form during storage is not desirable because this may cause instabilities when an API is embedded in the lipid particles. To identify potential changes in the polymorphic form of the lipid nanoparticles, samples of the suspension and ODFs (lipid content 0.29, formulation HPMC/SDS) were stored for six months at 20 °C (40% relative humidity). The samples were analyzed every three months by DSC and the results of this stability study are shown in Figure 7.

Directly after manufacturing, almost all tristearin particles were in the α-polymorphic form in the suspension as well as the ODF. Over time, a clear shift in the melting temperature of the lipid particles in the suspension indicated a transformation from the metastable α- into the stable β-polymorph. The small melting event at approx. 61 °C detected mainly after a storage time of 6 months might indicate the presence of particles in the β’-polymorphic form in the suspension [28]. After 6 months, no particles in the metastable α-form remained in the suspension. 

A different behavior was observed for the ODFs. Here, the trend towards transition into the stable β-polymorph was much lower. After storage for 6 months, 88% of the particles still remained in the metastable α-polymorphic form. Apparently, the presence of a rigid film matrix instead of a liquid medium in the environment of the nanoparticles strongly retarded the polymorphic transformation. Accompanying particle size measurements on the suspension and the nanoparticles reconstituted by dissolving the ODFs confirmed that the respective particle sizes did not change significantly during storage.

### 3.4. Embedding of Different Types of Triglycerides in ODFs 

**Figure 8:**
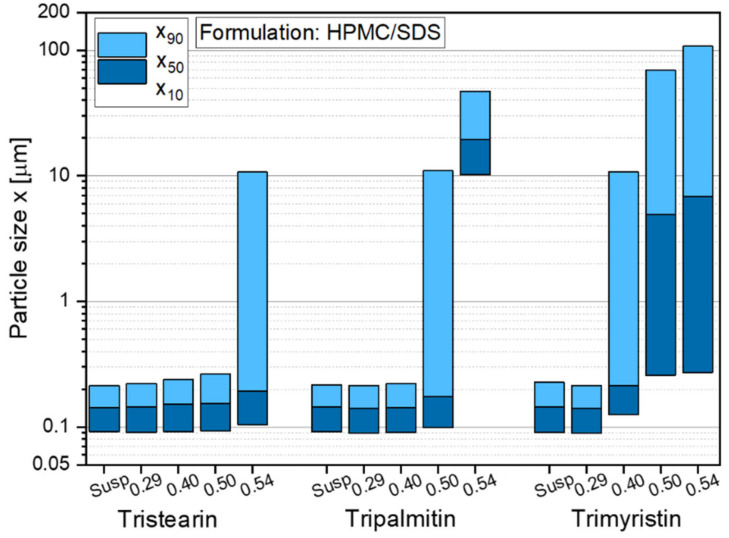
Particle size distribution of nanoparticles based on different triglycerides after redispersion from ODFs with lipid contents between 0.29 and 0.54 in comparison to the initial lipid suspensions (Susp). Data for tristearin from Figure 3.

The formulation screening experiments at the beginning of this study showed (see Figure 1) that besides tristearin, tripalmitin and trimyristin particles can also be stabilized with the chosen formulation HPMC/SDS. In order to investigate if these lipids could also be embedded in ODFs, films containing tripalmitin and trimyristin particles with different lipid contents were prepared. The sizes of the lipid nanoparticles after redispersion of the films in water are given in Figure 8. 

The results indicate that independent of the type of triglyceride lipid nanoparticles could be embedded in the film matrix without loss of their nanoparticulate properties but the maximum lipid content in the films depended on the type of embedded triglyceride. As shown before (Figure 3), a lipid content of up to 0.50 tristearin particles could be embedded in the ODFs without loss of their nanoparticle properties. Dependent on the fatty acid chain length of the triglycerides, the maximum possible lipid content constantly decreased to 0.40 for tripalmitin particles and 0.29 for trimyristin particles. The cause for these differences in the loading capacity of the films is currently under investigation. It will be evaluated whether the differences can result from the different polarities of the triglycerides used (due to varying fatty acid chain lengths), the particle shapes in terms of different aspect ratio (diameter/length) [43] or other chemical or physical characteristics.

## 4. Conclusions

Orodispersible films are a suitable delivery platform for solid lipid nanoparticles, as clearly shown in this study. The lipid nanoparticles could be redispersed from the film matrix with no loss of their nanoparticulate properties, while the films were of good quality in terms of mechanical properties and disintegration time. Furthermore, it was possible to stabilize the lipid particles in the metastable α-polymorphic form to a large extent over a storage time of at least 6 months when the tristearin suspension was stabilized with HPMC/SDS. 

The stabilizer screening yielded several appropriate particle formulations for the three triglycerides under investigation (tristearin, tripalmitin and trimyristin), with combinations of a polymer and the surfactant SDS as well as a formulation only with SDS being most promising. Closer investigations with tristearin indicated that the particle formulation has an impact on the film quality as well as on the redispersibility of the particles when the film matrix was dissolved in water. Two particle formulations (HPMC/SDS and SDS) were identified as particularly promising because they resulted in high lipid contents and good film qualities. For tristearin, lipid contents of up to 0.50 could be realized in the ODFs for the stabilization with HPMC/SDS and even up to 0.54 for the SDS-based formulation. While very high lipid contents in the films without loss of the nanoparticle properties could be realized when tristearin was embedded in the films, the maximum lipid load was clearly lower for the films loaded with tripalmitin (c_L,ODF_ = 0.40) and trimyristin (c_L,ODF_ = 0.29).

This study is considered a good starting point for further research on this topic. The embedding of solid lipid nanoparticles in a solid HPMC matrix is very promising and can have a stabilizing effect on the polymorphic state of the crystalline particles. Future work will focus, e.g., on the influence of the particles’ shape on their arrangement in the film matrix. Furthermore, it will be evaluated if the findings of this study can be transferred to API-loaded solid lipid nanoparticles. 

## Figures and Tables

**Table 1:** Influence of lipid content and particle formulation (HPMC/SDS and SDS) on the specific disintegration time, *n* = 3.

Lipid Content in ODFs (-)	Specific Disintegration Time (s/µm)	
	HPMC/SDS	SDS
0.29	0.56 ± 0.02	0.72 ± 0.08
0.40	1.06 ± 0.09	1.05 ± 0.10
0.50	1.29 ± 0.14	1.12 ± 0.20
0.54	1.43 ± 0.15	1.14 ± 0.11
0.57	1.58 ± 0.36	---
0.60	---	1.27 ± 0.11
0.64	---	0.55 ± 0.03
0.72	---	Not measurable

## Data Availability

Data is contained within the article.
